# Nurses’ Attitudes, Environmental Perceptions and Involvement in Research: A Multisite Study

**DOI:** 10.3390/nursrep15090344

**Published:** 2025-09-22

**Authors:** Amanda J. Hessels, Ulanda Marcus-Aiyeku, Mani Paliwal, Carrie Ann Catanzaro, Kimberly Dimino, Jessica Crowley, Jessica Miszlay, Maria Manzella, Kimkyla Kritch, Rachel Kilpatrick, Kim Kranz, Serpouhi S. Vartivarian, Barbara McGoey

**Affiliations:** 1 Ann May Center for Nursing Research, Hackensack Meridian Health, Tinton Falls, NJ 07712, USA; 2 Corporate Patient Safety and Quality, Hackensack Meridian Health, Edison, NJ 08837, USA; 3 Corporate Nursing Education, Hackensack Meridian Health, Neptune, NJ 07753, USA; 4 Southern Ocean University Medical Center, Hackensack Meridian Health, Manahawkin, NJ 08050, USA; 5 Ocean University Medical Center, Hackensack Meridian Health, Brick Township, NJ 08724, USA; 6 Jersey Shore University Medical Center, Hackensack Meridian Health, Neptune, NJ 07753, USA; 7 Riverview Medical Center, Hackensack Meridian Health, Red Bank, NJ 07701, USA; 8 Bayshore Medical Center, Hackensack Meridian Health, Holmdel, NJ 07733, USA; 9 Department of Nursing, Hackensack University Medical Center, Hackensack, NJ 07601, USA; 10 Hackensack University Medical Center, Hackensack Meridian Health Ryan White Foundation, Hackensack, NJ 07601, USA

**Keywords:** nursing research, nurse clinicians, research engagement, nursing education research, translational research, health services research, nursing administration research

## Abstract

**Background:** Although evidence-based practice is widely promoted in nursing, direct care nurses remain underrepresented in research activities. This study aimed to assess nurses’ attitudes toward research, their perceptions of the organizational research environment, and their levels of involvement, as well as identify key barriers and facilitators to engagement within a comprehensive healthcare system. This study also explored how racial and ethnic diversity within the nursing workforce may shape research engagement and contribute new perspectives to the field. **Methods:** A cross-sectional electronic survey was administered to registered nurses across 10 hospitals in a Northeast U.S. health system. The survey instrument assessed research attitudes, environment, involvement (past, present, future), and demographics. Descriptive and inferential statistics, including matched-pairs t-tests, were used to analyze responses. **Results:** Of 7655 invited nurses, 1094 responses were analyzed. Respondents were predominantly female (88.5%), White (56.8%), and employed full-time (87.1%) as clinical staff nurses (77.3%). While 54.8% had completed a formal research course (mainly within the past 1–3 years), informal research and statistics training were uncommon (17.4% and 5.4%, respectively). Nurses reported highly positive attitudes toward research (composite M = 2.15, SD = 0.51), especially its role in guiding practice, professional growth, and education. However, actual involvement was low. The most common current activities included practice change based on research (20.7%) and participation in committees (18.8%). Anticipated future engagement increased substantially, particularly in collaboration (+21.3%), committee participation (+20.6%), and IRB submission (+18.2%). The research environment was perceived as under-resourced, particularly in terms of protected time, funding, and mentorship. Statistically significant gaps were observed between perceived present and desired future supports (*p* < 0.01 for all 15 items). The Research Awareness Index revealed high rates of uncertainty about available resources (e.g., 66.1% did not know if internal funding existed). **Conclusions:** Nurses demonstrate strong positive attitudes and a desire to engage in research, including more advanced roles. Yet structural and informational barriers, particularly a lack of protected time, mentorship, and awareness of existing supports, limit participation. Investments in infrastructure, communication, and accessible development pathways are needed to translate nurses’ readiness into active research engagement. **Implications:** Institutions should prioritize making research support more visible and navigable while investing in mentorship, protected time, and user-friendly infrastructure. Addressing both facets will empower a highly motivated nursing workforce to engage in and lead practice-relevant research.

## 1. Introduction

Integrating research findings into clinical practice is vital for advancing healthcare and improving patient outcomes. Nurses, especially those providing direct bedside care, are ideally positioned to lead the integration of clinically relevant research into practice. However, nurses are often underrepresented in research endeavors, resulting in a critical gap in both the development and implementation of nursing science.

Barriers to nurse involvement in research are multifaceted. Individually, nurses encounter limited time, inadequate research training, and competing clinical responsibilities. Organizationally, these challenges are amplified by a lack of leadership support, insufficient funding, minimal mentorship, and few professional development opportunities. Advanced education is linked to greater engagement, yet even nurses with graduate degrees may lack practical skills or institutional support for sustained participation, highlighting the complex interplay between personal and organizational influences.

A substantial body of literature over several decades has chronicled these persistent barriers [1,2,3,4]. Recent studies affirm that these challenges continue, particularly in clinical settings where service delivery takes precedence over research involvement [5,6].

### 1.1. Theoretical Framework

Nursing science and organizational theory describe nurses’ attitudes, perceptions of work environments, and research involvement. The interplay of individual attitudes, organizational culture, and systemic support informs research participation.

Nurses’ attitudes and research involvement. Attitudes toward research are a consistent driver of engagement. Positive attitudes, often shaped by prior education and practical experience, predict increased willingness to take on research roles [7]. Confidence in applying research findings and recognizing their impact on patient outcomes further encourages participation [8]. Conversely, limited time, insufficient knowledge, and a lack of peer support restrict involvement [9].

Organizational environment. The context in which nurses work heavily influences their attitudes and engagement. Supportive organizations that offer dedicated research time, mentorship, leadership encouragement, and access to resources foster participation [10,11]. Such cultures also improve job satisfaction and commitment [12,13]. In contrast, high workloads, limited resources, or workplace incivility hinder engagement and contribute to burnout [14,15].

Interrelationships among concepts. Organizational settings shape attitudes, and positive attitudes, in turn, foster greater research involvement. Nurse leaders are crucial, as their support and modeling of research participation influence organizational culture [16]. Evidence-based interventions like journal clubs, training programs, and formal mentorship can enhance perceptions of support and promote research integration [10].

Theoretical foundations. Role theory and implementation science frameworks such as Promoting Action on Research Implementation in Health Services (PARIHS) and the Consolidated Framework for Implementation Research (CFIR) emphasize the importance of individual readiness, contextual support, and facilitation in adopting evidence-based practices [17,18]. Within these models, attitudes reflect individual preparedness, the organizational environment represents contextual support, and research involvement is the observable behavior. Positioning this study within these frameworks clarifies interpretation and underlines the importance of multi-level strategies for converting research readiness into active participation.

Implications for nursing science. Enhancing nurses’ research engagement requires both individual strategies (education, confidence-building) and systemic interventions (protected time, mentorship, leadership support). Broadening these supports can embed research in nursing culture and ultimately improve patient care outcomes [19].

### 1.2. Literature Review

Despite a growing focus on evidence-based practice, nurse engagement in research remains limited globally. While many studies examine factors influencing research participation in the U.S., international evidence shows that challenges and solutions are widespread.

Current research confirms that nurses generally hold positive attitudes toward research and see it as critical to quality care. Yet, self-assessed knowledge, confidence, and hands-on experience remain low, especially among bedside nurses and those in non-academic or resource-limited settings [20,21]. Studies from Europe, Asia, and Africa reinforce these findings, showing a widespread feeling among nurses of being unprepared or unsupported to participate actively in research [22,23,24].

Systematic reviews point to recurring barriers: heavy workloads, time constraints, insufficient training, lack of mentorship, and weak institutional support [25,26]. These obstacles are similar across various settings, from Magnet hospitals in the U.S. to tertiary institutions in Kenya, Italy, and Thailand [27,28,29,30,31,32]. Conversely, modifiable enablers include mentorship, ongoing education, protected time, and supportive organizational cultures [33,34].

Emerging literature highlights the importance of structural empowerment and psychological resources, optimism, self-efficacy, and resilience in promoting nurses’ engagement and professional growth, including research participation [35,36]. This underscores that engagement relies on both institutional resources and individual psychological factors.

Despite decades of inquiry, few interventions have been rigorously evaluated, and standardized tools for measuring engagement are rare, complicating cross-study comparisons. Frontline nurses from low- and middle-income countries are particularly underrepresented, and equity aspects such as gender, race, ethnicity, and geography are infrequently analyzed.

### 1.3. Purpose of the Study

This study seeks to address these gaps by exploring nurses’ attitudes, organizational environments, and barriers to research engagement in a large, diverse, multisite sample. This study was conducted within a large integrated health system in a Northeast U.S. state that includes 17 hospitals ranging in size from approximately 100 to nearly 1000 beds. The system comprises both academic/teaching centers and community hospitals, with a mix of Magnet- and non-Magnet–designated facilities. This diversity in size, type, and nursing infrastructure enhances the generalizability of the findings across different practice settings.

This work aligns with national priorities. The National League for Nursing 2023–2025 Research Priorities emphasize equity, innovation, well-being, and academic-practice partnerships [37]. Our study examines how organizational and educational supports affect engagement among clinical nurses. Similarly, the National Institute of Nursing Research 2022–2026 Strategic Plan prioritizes health equity, systems of care, and workforce development. By identifying institutional barriers to research participation, this study advances these priorities and informs research capacity-building strategies [38].

There remains an urgent need to foster practice environments that support and sustain nurse-led research. Strengthening research literacy and inquiry among nurses is essential for delivering high-quality, equitable care. This study aims to generate actionable insights to inform institutional strategies, as well as educational, structural, and cultural approaches, that can mitigate barriers and build capacity for nursing research. Our findings, situated in both national and global contexts, respond to calls for action by providing a nuanced understanding of how to support nurse engagement in the conduct of research.

## 2. Materials and Methods

### 2.1. Study Objectives

This cross-sectional study explores nurses’ perceptions of and experiences with barriers to research engagement. Specifically, the objectives are to:

1. Describe hospital-based nurses’ attitudes toward nursing research, their perceptions of the research environment, and their involvement in research activities (past, present, and future).
2. Identify organizational and structural factors associated with nurse research engagement.

By surveying a large and diverse healthcare organization, this study provides both locally actionable insights and broader contributions to the national conversation on building research capacity among frontline nurses.

### 2.2. Study Design and Setting

This study used a cross-sectional electronic survey design. The setting was a large, diverse healthcare system in a Northeast U.S. state. The hospitals varied, including both large and small facilities, as well as teaching and non-teaching hospitals, with different Magnet designation statuses, varying lengths of affiliation with the parent organization, and different levels of maturity in nursing research councils and infrastructure support. The system operates 17 hospitals, ranging from small community facilities (100–300 beds) to large academic medical centers (300–1000 beds). Of these, 4 are major academic/teaching hospitals and 13 are community hospitals, reflecting a broad mix of organizational contexts. The system includes Magnet-designated hospitals (≥5) and non-Magnet facilities, as well as institutions with varying lengths of affiliation within the parent organization and different levels of nursing research councils and infrastructure maturity. This heterogeneity provides a unique opportunity to examine nurse research engagement across a wide range of clinical environments, improving the relevance and transferability of the findings.

### 2.3. Instruments and Measures

A comprehensive 4-part questionnaire, adapted from Smirnoff et al. (2007), was used to collect data [3]. The instrument was adapted for electronic administration, and demographic questions were revised to reflect RN titles/roles and updated ethnicity categories and were positioned last on the survey. Extensive work was done on the survey build, converting paper forms to interactive web-based response interfaces using REDCap© (version 12.0.0) questionnaire and survey design technology [39,40]. REDCap© (Research Electronic Data Capture) is a secure, web-based software platform designed to support data capture for research studies, providing (1) an intuitive interface for validated data capture; (2) audit trails for tracking data manipulation and export procedures; (3) automated export procedures for seamless data downloads to common statistical packages; and (4) procedures for data integration and interoperability with external sources [39,40]. Several rounds of testing were conducted to ensure end-user engagement and clarity of intended response. Analysis was performed on the duration of completion and active user clicks to determine the technological difficulty of navigating between survey response types and various server displays (e.g., computer, tablet, cellphone). The survey was accessible via a hyperlink or QR code. The research team prioritized streamlining and usability to reduce respondent burden and attrition, thereby supporting survey participation from start to completion. The final version required approximately 9–15 min for completion and was noted on the recruitment materials.

The survey includes the following parts:

Part I measures attitudes towards nursing research and consists of 23 questions using a 5-point Likert scale from 1 (strongly agree) to 5 (strongly disagree) to assess RNs’ attitudes towards research (e.g., “I feel comfortable doing research”, “Nursing research is a pain in the neck”). The reported Cronbach’s alpha for this scale is 0.93; in this study, it was 0.92.

Part II of the survey assessed perceptions of the research environment using 15 items on a 4-point Likert scale from 1 (strongly agree) to 4 (strongly disagree). These items evaluated nurses’ perceptions and awareness of research support in their work setting, both as it currently exists and as it should be in the future. Example statements include: “Time is allowed during the workday for the writing/publishing of research”, “Nurses have qualified mentors for participating in research”, and “Consultation is available on the interpretation of nursing research findings”.

A Research Awareness Index (RAI) was created to summarize respondents’ knowledge and awareness of available research supports. “I don’t know” responses were treated as missing when calculating the mean score (to assess whether perceptions were generally positive or negative), then were used separately to compute the RAI as an indicator of lack of awareness. The reported internal consistency of this scale is high (Cronbach’s α = 0.94; in our study, α = 0.94). The conceptual basis for the RAI is that awareness is essential for engagement: nurses cannot act on opportunities they are unaware of. This aligns with organizational research culture frameworks and implementation science models like PARIHS and CFIR, which emphasize awareness/knowledge as key precursors to behavior change [10,17,18,41]. The index connects individual and organizational levels by reflecting both personal knowledge of research supports and how effectively institutions communicate about them. Therefore, the RAI offers a conceptually sound and practical measure of readiness for research involvement.

Part III measures research involvement using a 12-item dichotomous (“yes” or “no”) scale that asks whether respondents have participated in specific research activities in the past, are currently participating, or anticipate participating in the future. Example items include “Analyzing my research findings” and “Writing a proposal to conduct a nursing research study”. Reported Cronbach’s alpha values for the past, present, and future scales reported by Smirnoff et al. 2007 are 0.90, 0.93, and 0.96, respectively [3]. In our study, the values were 0.91, 0.91, and 0.97, respectively.

Part IV collected demographic data, including data such as gender, age, years of experience as a nurse, current position, highest academic degree, and prior research education.

### 2.4. Study Procedures and Sample

The study targeted registered nurses practicing in hospital settings within a health system, aiming for a 40% response rate from an estimated 6800 nurses using convenience sampling. After obtaining IRB approval and administrative support, the research was conducted over nine weeks in summer 2022, with no exclusion criteria, employing a multimodal recruitment strategy that included meetings and flyers with QR codes. An electronic survey was sent to 7655 nurses across 10 hospitals, resulting in 1439 responses, yielding an 18.8% response rate. Participants received a Certificate of Completion upon submitting the survey.

### 2.5. Analysis

IBM SPSS Statistics for Windows, version 22.0 (IBM Corp., Armonk, NY, USA) was used for statistical analysis [41]. Descriptive statistics were used to summarize demographic characteristics and responses to the survey items. To describe perceived barriers to conducting research among frontline nurses, data were analyzed at the individual respondent level, guided by the instrument domains (Attitudes, Environment, Involvement). Frequencies, percentages, means, and standard deviations were calculated as appropriate. Data were assessed for outliers and missing values. Different charts and graphs were used to visualize the data spread. A matched-pairs t-test was used to compare the means of each of the 15 item ratings for the present and future Research Environment scales. The significance level for all statistical tests was set at *p* < 0.05.

### 2.6. Ethical Considerations

This study was reviewed and approved by the health systems’ Institutional Review Board (Pro2021-1471). A waiver of documentation of informed consent was granted due to the minimal risk nature of the study and the anonymous data collection methods. Subjects were provided with information about their rights and risks, including that completing the survey was voluntary and anonymous, and that submitting the survey indicated their consent. All data collected were anonymous. No personally identifiable information or protected health information was collected. IP addresses were not recorded. Data collected through REDCap© (version 12.0.0) was downloaded and stored on password-protected and encrypted computers belonging to the investigators. Administrative data were entered and stored in the same secure manner.

## 3. Results

### 3.1. Response Rate and Demographics

The final analytic sample included 1094 registered nurses, the majority of whom were female (88.5%), White (56.8%), and employed full-time (87.1%) as clinical staff nurses (77.3%). Most held a baccalaureate degree (65.8%), with nearly 38% reporting over 20 years of nursing experience. While 54.8% had completed a formal research course, most within the past 1–3 years, only 17.4% reported any informal research training. Similarly, 64% had completed a formal statistics course, although nearly half of those had done so more than 8 years ago, and informal statistics training was rare (5.4%) (Table 1). These findings suggest an experienced nursing workforce with foundational academic exposure to research and statistics but limited ongoing or informal training.

### 3.2. Research Attitudes Results

The attitudes towards nursing research, as measured on a 1–4 Likert scale, indicate an overall positive attitude, as shown by the composite score (*M* = 2.15, *SD* = 0.51). The most positive attitude was towards nursing research being used to guide nursing practice (*M* = 1.42, *SD* = 0.56), followed by the positive attitude that nurses should have the opportunity to be involved in nursing research (*M* = 1.58, *SD* = 0.62). Only five of the 23 items had a mean rating greater than 2.5, indicating a negative perception, most notably hearing the results of nursing studies (*M* = 3.25, *SD* = 1.01), followed by identifying clinical problems that need to be researched (*M* = 2.81, *SD* = 1.04). The remaining items, shown in ascending order from most to least positive, are shown in Table 2.

### 3.3. Research Environment Results

Regarding the Research Environment, mean scores lower than 2.4 reflect positive perceptions of the present environment, whereas those higher than 2.5 indicate negative perceptions. Ratings indicate negative perceptions of six of the items (Table 3). There was a statistically significant difference between present and future for each item by paired sample T-test, where the desired future support was higher than the present support ratings. The Research Awareness Index (RAI) was calculated to provide greater insight into perceptions of the environment by identifying the proportion of respondents who neither had a positive nor negative perception; they were unaware and did not know if a support characteristic was present in their work environment (Table 4).

### 3.4. Research Involvement Results

**Figure 1:**
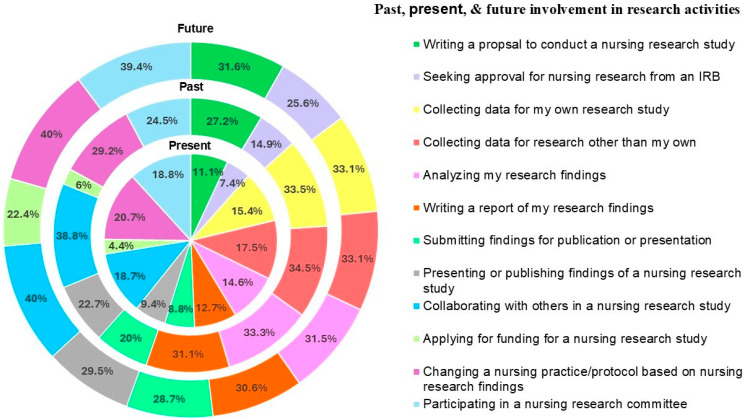
Involvement in Research Activities.

Nurses were asked about their involvement in research activities in the past, present, and anticipated future. Nurses reported less involvement in the present than in both the past and the desired future involvement, as shown in Figure 1. The items with the highest involvement reported in the past were “Collaborating with others in nursing research study” (38.8%) and “Collecting data for research other than my own” (34.5%). The items with the highest percentage of responses to present involvement were “Changing a nursing practice/protocol based on nursing research findings” (20.7%) and “Participating in a nursing research committee” (18.8%). Looking toward future intended involvement, the highest percentages are in “Changing a nursing practice/protocol based on nursing research findings” (40%) and “Collaborating with others in a nursing research study” (40%). The most substantial increases in intended or anticipated involvement in research activities in the future compared to the present were seen in “Collaborating with others in nursing research study” (+21.3%), “Participating in a nursing research committee” (+20.6%), and “seeking IRB approval for a nursing research study” (+18.2%).

## 4. Discussion

This multisite study provides a detailed assessment of clinical nurses’ attitudes toward research, their current and anticipated involvement, and perceptions of the organizational environment in which research occurs. Findings suggest a workforce that is conceptually aligned with the goals of evidence-based and research-informed care, yet constrained by structural and contextual barriers. Nurses reported strong support for the role of research in practice and professional development, alongside a clear interest in expanding their participation, particularly in activities that reflect deeper engagement, such as collaboration and protocol development. These findings are consistent with recent calls from the National League for Nursing to strengthen academic–practice partnerships and cultivate research engagement through equity, innovation, and system-level support. However, key elements such as mentorship, protected time, and visibility of resources were both underdeveloped and poorly communicated in the present study.

The sections that follow examine these findings across three core domains: research attitudes, research environment, and research involvement. We also share insights on the diversity and representation of nurses in research and implementation science frameworks. Each highlights actionable opportunities to apply implementation strategies and inform system-level interventions that can accelerate nurse-led inquiry and strengthen the bridge between research and practice.

### 4.1. Research Attitudes

The overwhelmingly positive attitudes observed in this study challenge longstanding assumptions that bedside nurses are disinterested or disengaged from research. Consistent with previous literature demonstrating that nurses broadly value research and its role in improving patient outcomes, our findings reveal a powerful endorsement of research as foundational to nursing practice, education, and professional growth [20,42]. Importantly, nurses in this study expressed not only openness to research involvement but a desire to take on more active and complex roles—such as seeking IRB approval—countering earlier claims that such responsibilities are off-putting to clinical nurses [1,5,14,43].

This shift may reflect evolving norms in nursing education, the influence of evidence-based practice cultures, and a broader redefinition of the professional identity. Nonetheless, a pronounced gap persists between enthusiasm and actual engagement, suggesting that structural and organizational barriers, rather than lack of motivation, are the primary inhibitors. These results underscore the need to align institutional structures, resources, and research infrastructure with nurses’ demonstrated readiness. Beyond awareness, health systems must establish practical, accessible pathways to unlock the potential of a research-receptive workforce. In summary, our findings reinforce recent evidence that nurses strongly endorse the role of research in practice and professional growth, with many expressing interest in more complex activities (e.g., IRB applications, protocol development). This challenges older claims that bedside nurses avoid such roles and suggests a shift in professional identity toward seeing themselves as contributors to nursing science.

### 4.2. Research Environment

Data from the research environment reveal a disconnect between organizational aspirations and daily realities. Although nurses show strong interest in engagement, they perceive deficits in time, funding, mentorship, and access to consultation services, findings that align with previous studies documenting persistent barriers in clinical settings [4,5,15]. This study contributes by using a Research Awareness Index (RAI) that measures a less-explored aspect: how unaware nurses are of whether supports exist in their environment. Conceptually, the RAI is based on frameworks of organizational research culture and implementation science, where awareness is a crucial precursor to engagement. By assessing nurses’ familiarity with research processes, supports, and opportunities, the index provides a practical tool for identifying gaps, tracking progress, and guiding targeted efforts to increase research capacity. High rates of “I don’t know” responses across key areas (e.g., funding availability, consultation access) indicate that even when resources are available, they may be practically invisible to nurses. This builds on earlier work by highlighting not only the lack of support but also a breakdown in communication, visibility, and accessibility. Incorporating artificial intelligence (AI) could be a promising solution to improve visibility, accessibility, and support.

Compared with Smirnoff et al. (2007), who reported uniformly negative perceptions of the environment, our findings may indicate a modestly more optimistic workforce or institutional improvements over time—yet the gap between the present and desired future state remains [3]. Enhancing the visibility, navigability, and usability of infrastructure is thus as crucial as building capacity. For example, AI-enabled navigation tools could offer step-by-step guidance and real-time support to help nurses access policies, forms, mentorship, and consultation services [44,45]. Health systems should consider this a call to action to bridge both the practice–research and the support–awareness gaps.

### 4.3. Research Involvement

The progression from past to present to future involvement yields a key insight. Historically, nurses reported involvement clustered in collaborative and data-collection roles, consistent with literature characterizing nurses as research assistants rather than investigators or designers. In this study, nurses reported strong future interest in higher-order roles, including collaborating on studies (+21.3%), serving on research committees (+20.6%), and seeking IRB approval (+18.2%) [12,18]. This enthusiasm challenges earlier characterizations of reluctance tied to time constraints, confidence, or limited support [4,5,14,19].

The pattern of higher past engagement, lower present engagement, and optimistic future engagement also suggests a gap between readiness and reality. Historical participation may reflect legacy initiatives, more senior staff, or periods of more visible support, while current declines highlight structural and contextual barriers. Simultaneously, these findings indicate a shifting professional identity in which clinical nurses increasingly perceive themselves as contributors to nursing science rather than merely consumers of evidence. This evolution likely results from broader changes in nursing education, the expansion of academic–practice partnerships, and increased exposure to research within health system cultures that emphasize evidence-based practice [6,20]. The notable increase in interest in IRB engagement is particularly significant, considering its previous perception as burdensome or inaccessible to frontline clinicians. This rising willingness to move beyond observational roles toward research co-leadership emphasizes the necessity for targeted investment in mentorship, protected time, and structured participation pathways. Implementation and translational science strategies are apt to operationalize this alignment, establishing bidirectional pathways that allow nurses to both contribute to and utilize research in real-world contexts [21,46]. Addressing nurses in their current environment—motivated yet under-supported—presents a significant opportunity to advance nursing-led inquiry and to accelerate evidence-based improvements in patient care.

### 4.4. Diversity and Representation in Nursing Research Participation

Notably, the racial and ethnic composition of our sample diverges from national trends, with a greater proportion of non-White nurses than is typically reported in national workforce data, where approximately 80% of nurses identify as White (National Council of State Boards of Nursing) [47]. While this limits generalizability to all U.S. nurses, it also presents a valuable opportunity to generate new insights into research engagement among a more racially and ethnically diverse cohort.

National initiatives continue to emphasize the urgent need for a nursing workforce that reflects the diversity of the patient populations it serves. Yet disparities in representation persist, especially in academic and research roles (National Academies of Sciences, Engineering, and Medicine) [48]. Our findings suggest that racially and ethnically diverse nurses are not only present within clinical settings but also hold strong research attitudes and express interest in more substantive roles, contrary to assumptions that underrepresented groups are less engaged in research.

This highlights an important but underexplored area: the perspectives, contributions, and unique barriers faced by nurses of underrepresented groups in the research space. Future work should examine whether existing research infrastructures are equitably accessible and how support systems can be designed to engage and retain diverse clinicians in research. Prioritizing inclusive mentorship, culturally responsive training, and intentional strategies for representation can help strengthen nursing science and ensure that the evidence base reflects the full breadth of nursing practice. As the nursing profession continues to strive toward health equity and workforce diversity, capturing and understanding the experiences of diverse clinicians in research contexts becomes increasingly vital.

### 4.5. Linking to Implementation Science

The gap between nurses’ research readiness and their actual participation highlights a critical challenge in implementation. From a translational science perspective, these findings underscore the need for health systems to adopt systematic approaches to awareness-building, capacity development, and facilitation, embedding infrastructure that enables meaningful and sustainable nurse participation in research.

Implementation frameworks such as PARIHS and CFIR highlight the dynamic interaction of evidence, context, and facilitation. In this study, evidence (positive attitudes) was strong, and the contextual readiness was moderate, but facilitation—the active support that converts readiness into action—was limited. Addressing this requires multi-level strategies:

- Build capacity through structured training, research mentorship, and exposure to role models.
- Enhance visibility and navigation via centralized platforms, unit-level champions, and digital tools (e.g., AI-enabled guidance systems).
- Align organizational policies by embedding protected time, leadership advocacy, and integration of research into professional development frameworks.

Beyond these established frameworks, additional implementation science models highlight competencies and tools that can guide actionable change. The Implementation Science Competencies for Policy Transformation framework identifies skills essential for nurses to lead evidence-informed policy and practice transformations, including evidence appraisal, collaborative leadership, and data-driven decision-making [49]. The Interactive Systems Framework for Dissemination and Implementation and the Quality Implementation Tool provide practical guidance for planning, monitoring, and evaluating integration of innovations into routine practice [50]. These tools help operationalize readiness assessments and support continuous improvement cycles.

Importantly, implementation science distinguishes itself by focusing on processes that move from readiness to action [51]. Programs such as the Knowledge Translation Challenge have demonstrated how structured training and support can empower nurses to lead change initiatives and embed evidence-based practices within their clinical settings [52]. Similarly, assessments of organizational readiness for evidence-based practice can guide tailored interventions to minimize barriers and enhance facilitators, creating cultures where EBP is supported and sustained [53,54].

Finally, the Research Awareness Index (RAI) introduced in this study contributes a novel implementation lens by operationalizing awareness as a necessary precursor to engagement. Conceptually grounded in organizational research culture and implementation science frameworks, the RAI quantifies nurses’ familiarity with research processes, supports, and opportunities. In doing so, it captures the intermediate step between positive attitudes and actual involvement, highlighting where communication, training, or mentorship may fall short. Tracking the RAI over time can guide targeted interventions—such as orientation modules or awareness campaigns—benchmark progress across sites and reveal inequities in access to research opportunities. In this way, the RAI provides a bridge between conceptual readiness frameworks and actionable strategies to strengthen research capacity at both the individual and system level, directly aligning with national research priorities (e.g., NLN 2023–2025; NINR 2022–2026) that call for building nursing research capacity and advancing equitable workforce participation.

Taken together, these frameworks and tools provide a roadmap for translating positive attitudes into meaningful research engagement. By coupling strong individual readiness with deliberate organizational facilitation, health systems can bridge the readiness–reality gap and accelerate nursing-led inquiry and evidence-based improvements in patient care.

### 4.6. Limitations

This study has several limitations. The cross-sectional, self-reported design precludes causal inference and may introduce recall or social desirability bias. The overall 18.8% response rate raises concerns of nonresponse bias. Systematic differences may exist between respondents and non-respondents; for example, nurses who chose to participate may have a greater interest in research or distinct workplace experiences compared to those who did not respond. These factors could influence the representativeness and generalizability of our findings, and the low response rate itself may also signal broader disengagement from research among some nurses. As a result, perspectives of less-involved nurses, who may face the most significant barriers, may be underrepresented.

At the same time, the study benefitted from deliberate strategies designed to mitigate these challenges. The survey was carefully adapted for electronic administration using REDCap© technology, with extensive testing to ensure clarity, usability, and accessibility across devices. Several rounds of pilot testing analyzed completion time and click patterns to reduce burden, improve navigation, and minimize attrition. The final instrument required only 9–15 min for completion, balancing comprehensiveness with feasibility. In addition, grassroots outreach, including unit-level recruitment with Chief Nursing Officer/Executive support and multiple modes of access (QR code, hyperlink), was deliberately incorporated to broaden participation. These features do not eliminate concerns about representativeness, but they strengthen confidence in the validity of the responses collected. Thus, while the response rate remains a limitation, the extensive design and outreach efforts provide confidence that the findings reflect meaningful perspectives across a large, diverse nursing workforce.

Respondents in this study were highly educated (65% bachelor’s, nearly 20% master’s/doctoral) and more racially and ethnically diverse than national averages. While this may limit generalizability, it also provides valuable insights into the perspectives and readiness of racially and ethnically diverse nurses to participate in research. The modest, non-monetary “Certificate of Completion” offered upon survey completion may also have influenced participation patterns and is acknowledged as a potential source of response bias.

Analyses were limited to the nurse level; hospital-level factors such as Magnet status, leadership culture, or infrastructure maturity were not examined but may shape engagement. Finally, while this study examined the interrelationships among nurses’ attitudes, perceptions of the organizational environment, and their research involvement, it did not fully capture all components emphasized in implementation science frameworks such as PARIHS or CFIR. Specifically, the construct of facilitation, the active processes and supports that enable translation of readiness into action, was not directly measured. As such, although our findings illuminate important associations between attitudes and environment, they may underrepresent the role of facilitation in shaping actual research engagement. Future studies should consider a more comprehensive, multi-level assessment across these domains to strengthen both theoretical alignment and practical applicability.

## 5. Conclusions

This study shows that while nurses are attitudinally ready to engage in research, they remain structurally under-supported. Positive attitudes (evidence) were strong, but contextual readiness was moderate, and facilitation was limited, consistent with implementation science frameworks such as PARIHS and CFIR. Bridging this gap requires capacity-building (training and mentorship), visibility of resources, and alignment of organizational policies (e.g., protected time, leadership advocacy). The Research Awareness Index (RAI) offers a practical tool for identifying gaps, tracking progress, and guiding targeted interventions. Coupling individual readiness with deliberate organizational support can close the aspiration–action gap and accelerate nurse-led inquiry to improve patient care.

## 6. Implications and Actionable Strategies for Practice, Administration, and Research

**Figure 2:**
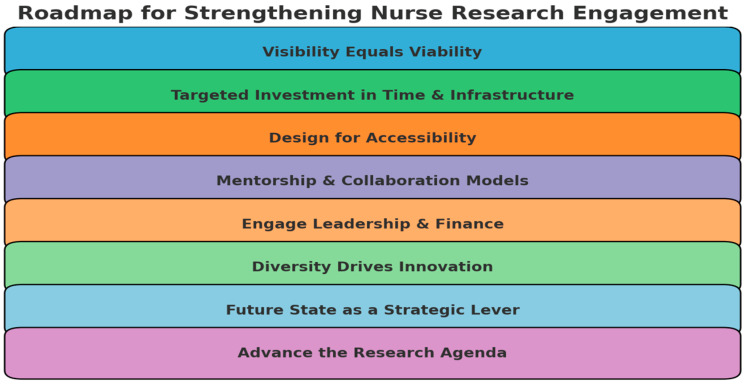
Roadmap for Research Engagement.

Nurses are attitudinally ready but structurally under-supported to engage in and conduct research. To close this readiness–reality gap, health systems must move beyond valuing research in principle to creating conditions where frontline nurses can directly participate in study design, data collection, analysis, and dissemination. This requires making resources visible, ensuring equitable access, protecting time, embedding mentorship, and aligning institutional priorities with nurses’ aspirations. The following strategies provide a roadmap for translating these implications into action (Figure 2).

- Visibility Equals Viability
  If nurses are unaware of existing research supports, it is functionally equivalent to them not existing. Organizations should prioritize communication, navigation tools, and unit-level champions to ensure that research opportunities are not only visible but also explicitly invite staff nurses to participate as collaborators, investigators, and co-authors.
- Targeted Investment in Time and Infrastructure
  Substantial deficits exist in the time afforded for research. Strategies include piloting protected “research hours” within clinical schedules, developing hybrid clinical–research roles for advanced-degree bedside nurses, and creating micro-grants to support staff-led projects. These investments signal that nurse-led inquiry is both possible and valued.
- Design for Accessibility
  Centralize funding databases, mentorship directories, IRB templates, and statistical consultation services in searchable, nurse-friendly platforms. Emerging innovations, such as AI-enabled navigation systems, can further reduce barriers by guiding nurses through the research process from idea generation to dissemination.
- Mentorship and Collaboration Models
  Pair bedside nurses with experienced nurse scientists to co-develop abstracts, IRB applications, study protocols, and manuscripts. Embedding mentorship within unit-based councils or Magnet structures ensures that opportunities to conduct and disseminate research are visible and equitably available across all units.
- Engage Leadership and Finance
  Strengthen leadership involvement by highlighting the return on investment of nurse-led research for workforce retention, quality improvement, and Magnet recognition. Finance and nursing leaders can partner to sustain infrastructure through joint appointments, shared funding with academic institutions, and resource pooling across facilities.
- Diversity Drives Innovation
  Broad demographic and clinical representation in nursing research enhances both equity and innovation. The Research Awareness Index can help identify groups with lower awareness or engagement, allowing for targeted strategies—such as culturally responsive training, inclusive mentorship, and journal clubs tailored to early-career or underrepresented nurses—to foster more diverse research participation.
- Future State as a Strategic Lever
  Nurses’ strong desire for greater involvement in research should be used as a catalyst for organizational change, particularly in Magnet institutions or systems seeking to promote evidence-based care. Aligning professional development pathways with opportunities to *conduct research*—from protocol writing to dissemination—can accelerate both research capacity and system innovation.
- Advance the Research Agenda
  Future studies should evaluate training models that support bedside nurses as investigators, test hybrid clinical–research roles, and explore underrepresented voices through qualitative and longitudinal designs. Building this evidence base will refine infrastructure and ensure sustainable, system-wide approaches to nurse research engagement.

## Figures and Tables

**Table 1:** Demographic Characteristics (*N* = 1094).

Demographic Characteristics	Values	*n* (%)
Gender (*n* = 953)	Female	843 (88.5%)
	Male	73 (7.7%)
	Prefer not to answer	37 (3.8%)
Ethnicity (*n* = 960)	White	545 (56.8%)
	Asian	194 (20.2%)
	Hispanic or Latino	95 (9.9%)
	Black or African American	74 (7.7%)
	Other/Prefer not to answer	52 (5.4%)
Age range (*n* = 958)	>=60 yrs	133 (13.9%)
	55–59	119 (12.4%)
	45–54	217 (22.7%)
	35–44	208 (21.7%)
	25–34	243 (25.3%)
	<25 yrs	38 (4%)
Years as nurse (*n* = 964)	>20 yrs	362 (37.6%)
	16–20 yrs	103 (10.7%)
	11–15 yrs	111 (11.5%)
	6–10 yrs	147 (15.2%)
	3–5 yrs	89 (9.2%)
	<3 yrs	152 (15.8%)
Employment status (*n* = 967)	Full-time	842 (87.1%)
	Part-time	96 (9.9%)
	Per Diem	29 (3%)
Position/Job Title (*n* = 948)	Clinical Staff Nurse	733 (77.3%)
	Clinical Nurse Manager	77 (8.1%)
	Nursing Administrator	47 (5%)
	Other	91 (9.6%)
Shift worked (*n* = 963)	Day	647 (67.2%)
	Night	242 (25.1%)
	Evening	46 (4.8%)
Highest nursing degree (*n* = 966)	Doctorate	30 (3.1%)
	Masters	159 (16.5%)
	Baccalaureate	636 (65.8%)
	Associate/Diploma	141 (14.5%)
Current enrollment in a degree program (*n* = 270)	Doctorate	36 (13.3%)
	Masters	138 (51.1%)
	Baccalaureate	95 (35.2%)
Completed a formal (academic) research course (*n* = 965)	Yes	529 (54.8%)
	No	436 (45.2%)
When did you complete the research course (*n* = 517)	Within past 1–3 yrs	242 (46.8%)
	4–5 yrs	88 (17%)
	6–7 yrs	49 (9.5%)
	More than 8 yrs	138 (26.7%)
Completed any informal research training (*n* = 959)	Yes	167 (17.4%)
	No	792 (82.6%)
Completed a formal (academic) statistics course (*n* = 963)	Yes	616 (64%)
	No	347 (36%)
When did you complete the statistics course (*n* = 600)	Within past 1–3 yrs	107 (17.8%)
	4–5 yrs	126 (21%)
	6–7 yrs	80 (13.3%)
	More than 8 yrs	287 (47.8%)
Completed any informal statistics training (*n* = 949)	Yes	51 (5.4%)
	No	898 (94.6%)

Note: Categories may not total *N* = 1094 due to missing data.

**Table 2:** Attitudes towards Nursing Research (*N* = 1094).

Statement (Item #)	*Mean*	*SD*
Nursing research findings should guide nursing practice. (1)	1.42	0.56
Nurses should have the opportunity to be involved in nursing research. (8)	1.58	0.62
The use of clinical nursing research findings will improve the quality of nursing care. (16)	1.62	0.63
Nursing research findings should be used as a foundation for nursing practice and education. (13)	1.65	0.64
Nursing interventions should be based on clinical nursing research findings. (17)	1.65	0.63
Engaging in research contributes to my professional growth. (7)	1.69	0.68
I feel that when I am taking part in nursing research, I am contributing to the science of nursing. (9)	1.69	0.70
Participating in nursing research is a valuable learning experience. (6)	1.71	0.64
Participating in nursing research increases my research skills. (15)	1.77	0.66
I am willing to collaborate in nursing research. (14)	1.93	0.77
I can engage in nursing research that is in my area of interest. (3)	2.06	0.79
If I could, I would help conduct a nursing research study about a clinical problem. (23)	2.23	0.90
Participating in nursing research makes me want to learn more about the research process. (20)	2.27	0.90
I would like to study a clinical problem. (12)	2.29	0.90
I keep informed about nursing research findings through journals and conferences. (19)	2.36	0.95
I gain little when I take part in a nursing study. (22 *)	2.42	1.00
I feel comfortable doing research. (2)	2.46	0.94
I like to do nursing research. (10)	2.49	0.99
Discussing how to use research findings is boring. (18 *)	2.61	1.03
Nursing research is a “pain in the neck”. (4 *)	2.62	0.99
If nursing research must be done, I hope someone else will do it. (5 *)	2.72	1.03
I have identified a clinical problem that should be researched. (21)	2.81	1.04
I seldom hear the results of nursing studies. (11 *)	3.25	1.01
**Composite Perceptions Score**	**2.15**	**0.51**

Notes: Items rated on a 5-point Likert scale from 1 (strongly agree) to 5 (strongly disagree). Items are ordered in ascending order from most positive to least positive; “*” indicates items were reverse-coded.

**Table 3:** Difference in Means of Nurse Perceptions of Support for Research: Present and Future Environment (*N* = 1094).

Item	Sample Size (*N*)	Presently Exists *Mean* (*SD*)	Future Support *Mean* (*SD*)	*p*-Value (Sig. < 0.05)
Time is allowed during the workday for the writing/publishing of research	696	3.23 (0.90)	2.46 (1.08)	0.000 *
On duty time is permitted for proposal writing.	523	3.12 (0.85)	2.43 (1.01)	0.000 *
On duty time is allowed for analysis of nursing research findings.	507	3.02 (0.87)	2.32 (0.97)	0.000 *
Nurses are encouraged to seek outside funding for research.	436	2.33 (0.85)	2.1 (0.87)	0.000 *
Information about research funding is available.	379	2.52 (0.83)	1.98 (0.74)	0.000 *
Monies from internal resources are available for nursing research.	191	2.57 (0.82)	1.97 (0.77)	0.000 *
Time is given to attend nursing research conferences.	662	2.43 (0.88)	1.96 (0.85)	0.000 *
Consultation services for nursing research projects are available.	471	2.32 (0.83)	1.92 (0.74)	0.000 *
Funds for nursing research projects are available.	276	2.5 (0.82)	1.91 (0.72)	0.000 *
Reviewers are available to evaluate the scientific merit of nursing research projects.	496	2.16 (0.78)	1.87 (0.68)	0.000*
Consultation is available on the interpretation on the nursing research finding.	404	2.17 (0.73)	1.82 (0.64)	0.000 *
Nurses with established research skills are available for consultation.	492	2.12 (0.72)	1.8 (0.62)	0.000 *
Nurses have qualified mentors for participating in research	656	2.17 (0.78)	1.79 (0.68)	0.000 *
Nursing administration encourages nurses to present research findings at conferences.	670	2.06 (0.75)	1.76 (0.63)	0.000 *
Nursing administration supports nursing research.	678	1.99 (0.73)	1.75 (0.66)	0.000 *

Notes: The scale includes 15 items rated on a 4-point Likert scale from *strongly agree* (1) to *strongly disagree* (4), where lower values indicate stronger agreement. A matched pairs t-test was performed; “*” indicates a significant difference at *p* < 0.05. The table is sorted in descending order of “future” responses.

**Table 4:** Research Awareness Index (RAI): Nurse Perceptions of Present Environment of Support (*N* = 1094).

Item	Number of “I Don’t Know” Responses	RAI (% Total Responses Marked “I Don’t Know”)
Funds for nursing research projects are available.	646	66.1%
Monies from internal resources are available for nursing research.	631	65.1%
Information about research funding is available.	538	54.7%
Consultation is available on the interpretation on the nursing research finding.	507	51.9%
Nurses are encouraged to seek outside funding for research.	497	48.5%
Consultation services for nursing research projects are available.	448	45.3%
Reviewers are available to evaluate the scientific merit of nursing research projects.	422	42.8%
Nurses with established research skills are available for consultation.	403	41.0%
On duty time is allowed for analysis of nursing research findings.	358	36.7%
On duty time is permitted for proposal writing.	360	36.2%
Nurses have qualified mentors for participating in research	263	25.7%
Nursing administration encourages nurses to present research findings at conferences.	223	22.5%
Time is given to attend nursing research conferences.	209	21.1%
Nursing administration supports nursing research.	195	20.0%
Time is allowed during the workday for the writing/publishing of research	176	17.5%

Notes: Research Awareness Index (RAI) calculated as % total responses to items marked “I don’t know”. Items are ordered from least to most aware.

## Data Availability

The original contributions presented in this study are included in the article. Further inquiries can be made to the corresponding author(s).

## Public Involvement Statement

No public involvement in any aspect of this research.

## Guidelines and Standards Statement

This manuscript was drafted using the STROBE guidelines for cross-sectional studies by von Elm E, Altman DG, Egger M, Pocock SJ, Gotzsche PC, and Vandenbroucke JP. Strengthening the Reporting of Observational Studies in Epidemiology (STROBE) Statement: guidelines for reporting observational studies (2007).

## References

[1] Funk S.G., Tornquist E.M., Champagne M.T. (1995). Barriers and facilitators of research utilization: An integrative review. Nurs. Clin. N. Am..

[2] Rizzuto C., Bostrom J., Suter W.N., Chenitz W.C. (1994). Predictors of nurses’ involvement in research activities. West. J. Nurs. Res..

[3] Smirnoff M., Ramirez M., Kooplimae L., Gibney M., McEvoy M.D. (2007). Nurses’ attitudes toward nursing research at a metropolitan medical center. Appl. Nurs. Res..

[4] Hagan J., Walden M. (2017). Development and evaluation of the barriers to nurses’ participation in research questionnaire. Clin. Nurs. Res..

[5] Breimaier H.E., Halfens R.J., Lohrmann C. (2011). Nurses’ wishes, knowledge, attitudes and perceived barriers on implementing research findings into practice among graduate nurses in Austria. J. Clin. Nurs..

[6] Edwards N., Kahwa E., Webber J., Roelofs S., Mill J. (2009). Building capacity for nurse-led research. Int. Nurs. Rev..

[7] Wells N., Baggs J.G. (1994). A survey of practicing nurses’ research interests and activities. Clin. Nurse Spec..

[8] Roxburgh M. (2006). An exploration of factors which constrain nurses from research participation. J. Clin. Nurs..

[9] Fink R., Thompson C.J., Bonnes D. (2005). Overcoming barriers and promoting the use of research in practice. J. Nurs. Adm..

[10] Seren Intepeler S., Esrefgil G., Yilmazmis F., Bengu N., Gunes Dinc N., Ileri S., Ataman Z., Dirik H.F. (2019). Role of job satisfaction and work environment on the organizational commitment of nurses: A cross-sectional study. Contemp. Nurse.

[11] Cumbey D.A., Alexander J.W. (1998). The relationship of job satisfaction with organizational variables in public health nursing. J. Nurs. Adm..

[12] Leiter M.P., Spence Laschinger H.K. (2006). Relationships of work and practice environment to professional burnout. Nurs. Res..

[13] Wu Y., Wang J., Liu J., Zheng J., Liu K., Baggs J.G., Liu X., You L. (2020). The impact of work environment on workplace violence, burnout and work attitudes for hospital nurses: A structural equation modelling analysis. J. Nurs. Manag..

[14] Lee S., Gifford J., Flood V. (2024). Enablers and Barriers of Research Engagement Among Clinician Researchers: Nursing, Allied Health and Medical Professionals. J. Multidiscip. Healthc..

[15] Bonner A., Sando J. (2008). Examining the knowledge, attitude and use of research by nurses. J. Nurs. Manag..

[16] Rycroft-Malone J. (2004). The PARIHS framework—A framework for guiding the implementation of evidence-based practice. J. Nurs. Care Qual..

[17] Damschroder L.J., Aron D.C., Keith R.E., Kirsh S.R., Alexander J.A., Lowery J.C. (2009). Fostering implementation of health services research findings into practice: A consolidated framework for advancing implementation science. Implement. Sci..

[18] Hetland B., Hickman R., McAndrew N., Daly B. (2017). Factors Influencing Active Family Engagement in Care Among Critical Care Nurses. AACN Adv. Crit. Care.

[19] Drury A., Fessele K.L., Robson P., Law E., Barton-Burke M., Thom B. (2024). Exploring research engagement among nurses in a Magnet^®^-recognized cancer center: An analysis of knowledge, attitudes, practices, and influencing factors. Asia-Pac. J. Oncol. Nurs..

[20] Alqahtani N., Oh K.M., Kitsantas P., Rodan M. (2020). Nurses’ evidence-based practice knowledge, attitudes and implementation: A cross-sectional study. J. Clin. Nurs..

[21] Olade R.A. (2003). Attitudes and factors affecting research utilization. Nurs. Forum.

[22] Gundo R., Mulaudzi F., Lavhelani R., Koloti M., Moasa P. (2025). Clinical nurses’ perceptions of research in Gauteng Province, South Africa: A qualitative study. BMC Nurs..

[23] Šatara S.S., Stojaković N., Jelić A.G., Maksimović Ž.M., Bojić M.G., Tepić S.P. (2023). Knowledge, attitudes and nursing self-evaluation related to clinical research. Scr. Medica.

[24] Scarsini S., Narduzzi B., Cadorin L., Palese A. (2022). Perceived Barriers and Enablers of Nursing Research in the Italian Context: Findings from a Systematic Review. Slov. J. Public Health.

[25] Smith S., Johnson G. (2023). A systematic review of the barriers, enablers and strategies to embedding translational research within the public hospital system focusing on nursing and allied health professions. PLoS ONE..

[26] Kaseka P.U., Mbakaya B.C. (2022). Knowledge, attitude and use of evidence based practice (EBP) among registered nurse-midwives practicing in central hospitals in Malawi: A cross-sectional survey. BMC Nurs..

[27] Kassie M., Tadele A., Beza L., Adal O., Azazh A. (2025). Evidence-based practice utilization and associated factors among nurses in the emergency department of selected public hospitals, Addis Ababa, Ethiopia, 2024: Cross-sectional study. BMC Health Serv. Res..

[28] De Marinis M.G., Piredda M., Pascarella M.C., Vincenzi B., Spiga F., Tartaglini D., Matarese M. (2022). Barriers and facilitators of nursing research in Italy: A systematic review. Slov. J. Public Health.

[29] Bressan V., Bagnasco A., Bianchi M., Rossi S., Moschetti F., Barisone M., Pellegrini R., Aleo G., Timmins F., Sasso L. (2017). Barriers to research awareness among nurses in Italy. J. Nurs. Manag..

[30] Srisuphan W., Sawaengdee K. (2012). Research in nursing and midwifery in Thailand: Growth and challenges. Nurs. Health Sci..

[31] Saunders H., Vehviläinen-Julkunen K. (2017). The state of readiness for evidence-based practice among nurses: An integrative review. Int. J. Nurs. Stud..

[32] Matagira-Rondón G., Agudelo-Cifuentes M.C., Toupin I., Bergeron D.A. (2022). Nursing research in Latin America: Priorities and possible solutions to move it forward. Rev. CES Enferm..

[33] Chien W.T., Bai Q., Wong W.K., Wang H., Lu X. (2013). Nurses’ perceived barriers to and facilitators of research utilization in mainland China: A cross-sectional survey. Open Nurs. J..

[34] Poku C.A., Bayuo J., Agyare V.A., Sarkodie N.K., Bam V. (2025). Work engagement, resilience and turnover intentions among nurses: A mediation analysis. BMC Health Serv. Res..

[35] Zhang Y., Qiu R., Wang Y., Ye Z. (2025). Navigating the future: Unveiling new facets of nurse work engagement. BMC Nurs..

[36] National League for Nursing (2024). NLN Research Priorities in Nursing Education 2023–2025.

[37] National Institute of Nursing Research (2024). Strategic Plan 2022–2026: Advancing Nursing Research for a Healthier Nation.

[38] Harris P.A., Taylor R., Thielke R., Payne J., Gonzalez N., Conde J.G. (2009). Research electronic data capture (REDCap)—A metadata-driven methodology and workflow process for providing translational research informatics support. J. Biomed. Inform..

[39] Harris P.A., Taylor R., Minor B.L., Elliott V., Fernandez M., O’Neal L., McLeod L., Delacqua G., Delacqua F., Kirby J. (2019). The REDCap consortium: Building an international community of software partners. J. Biomed. Inform..

[40] Bostrom A.C., Malnight M., MacDougall J., Hargis D. (1989). Staff nurses’ attitudes toward nursing research: A descriptive survey. J. Adv. Nurs..

[41] IBM Corp (2020). IBM SPSS Statistics for Windows.

[42] Hagan J. (2018). Nurse satisfaction with opportunities to engage in research. West. J. Nurs. Res..

[43] Funk S.G., Champagne M.T., Wiese R.A., Tornquist E.M. (1991). Barriers to using research findings in practice: The clinician’s perspective. Appl. Nurs. Res..

[44] Yasin Y.M., Al-Hamad A., Metersky K., Kehyayan V. (2025). Incorporation of artificial intelligence into nursing research: A scoping review. Int. Nurs. Rev..

[45] Ramadan O.M.E., Alruwaili M.M., Alruwaili A.N., Elsehrawy M.G., Alanazi S. (2024). Facilitators and barriers to AI adoption in nursing practice: A qualitative study of registered nurses’ perspectives. BMC Nurs..

[46] Rojaye J.O., Netangaheni R.T. (2023). Participation of nurses in research development. Health SA Gesondheid.

[47] National Council of State Boards of Nursing (2022). 2022 National Nursing Workforce Survey. J. Nurs. Regul..

[48] National Academies of Sciences, Engineering, and Medicine (2021). The Future of Nursing 2020–2030: Charting a Path to Achieve Health Equity.

[49] Al-Moteri M., Aljuaid J., Alqurashi H.M., Otayni M.M., Al-Jaid M.H., Ahmed A.M.H., Sufyani B.O.A., Almalki S.A., Cagoco A.D., Bamansur R.M. (2025). Implementation Science Competencies for Policy Transformation Framework (ISCPT). Healthcare.

[50] Meyers D.C., Katz J., Chien V., Wandersman A., Scaccia J.P., Wright A. (2012). Practical implementation science: Developing and piloting the Quality Implementation Tool. Am. J. Community Psychol..

[51] Wandersman A., Duffy J., Flaspohler P., Noonan R., Lubell K., Stillman L., Saul J. (2008). Bridging the gap between prevention research and practice: The interactive systems framework for dissemination and implementation. Am. J. Community Psychol..

[52] Chisholm A.E., Russolillo A., Carter M., Steinberg M., Lambert L., Knox A., Black A., Hoens A. (2024). Advancing evidence-based practice through the Knowledge Translation Challenge: Nurses’ important roles in research, implementation science and practice change. J. Adv. Nurs..

[53] Thiel L., Ghosh Y. (2008). Determining registered nurses’ readiness for evidence-based practice. Worldviews Evid.-Based Nurs..

[54] Iwama K., Travis A., Nowlin S., Souffront K., Finlayson C., Gorbenko K., Cohen B. (2023). Barriers, facilitators, and opportunities for Doctor of Nursing Practice engagement in translational research. Nurs. Outlook.

